# Comparative analysis of the CO_2_ emissions of expressway and arterial road traffic: A case in Beijing

**DOI:** 10.1371/journal.pone.0231536

**Published:** 2020-04-14

**Authors:** Ji Zheng, Suocheng Dong, Yingjie Hu, Yu Li

**Affiliations:** 1 Institute of Geographic Sciences and Natural Resources Research, Chinese Academy of Sciences, Beijing, China; 2 University of Chinese Academy of Sciences, Beijing, China; 3 College of City Construction, Jiangxi Normal University, Nanchang, China; Central South University, CHINA

## Abstract

Urban traffic is an important source of global CO_2_ emissions. Uncovering the temporal and structural characteristics can provide scientific support to identify the variation regulation and main subjects of urban traffic CO_2_ emissions. The road class is one of the most important factors influencing the urban traffic CO_2_ emissions. Based on the annual traffic field monitoring work in 2014 and the localized MOVES model, this study unravels the temporal variation and structural characteristics of the urban traffic CO_2_ emissions and conducts a comparative analysis of expressway (5R) and arterial road (DB), two typical classes of urban roads in Beijing. Obvious differences exist in the temporal variation characteristics of the traffic CO_2_ emissions between the expressway and arterial road at the annual, week and daily scales. The annual traffic CO_2_ emissions at the expressway (5R, with 47271.15 t) are more than ten times than those of the arterial road (DB, with 4139.19 t). Stronger weekly “rest effect” is observed at the expressway than the arterial road. The daily peak time and duration of the traffic CO_2_ emissions between the two classes of urban roads show significant differences particular in the evening peak. The differences of the structural characteristics between the two classes of urban roads are mainly reflected on the contribution of the public and freight transportation. Passenger vehicles play a predominant role at both the two classes of urban roads. The public transportation contributed more at DB (24.76%) than 5R (5.47%), and the freight transportation contributed more at 5R (23.41%) than DB (3.49%). The results suggest that the influence of traffic CO_2_ emissions on the CO_2_ flux is significant at the residential and commercial mixed underlying urban areas with arterial roads (DB) but not significant at the underlying urban park area with expressway (5R) in this study. The vegetation cover in urban areas have effects on the CO_2_ reduction. Increasing the design and construction of the green space along the urban roads with busy traffic flow will be an effective way to mitigate the urban traffic CO_2_ emissions and build the low-carbon cities.

## Introduction

Urban areas account for more than 70% of the global fossil fuel CO_2_ emissions [1]. To provide scientific references for urban planning decisions on CO_2_ reductions at a finer scale, direct CO_2_ flux measurements by the eddy covariance (EC) have been widely applied in urban areas [2–4]. More than 40 urban CO_2_ flux sites have joined the Urban Flux Network (http://ibis.geog.ubc.ca/urbanflux/). Traffic, contributing 23% of total CO_2_ emissions [5], is one of the most important direct CO_2_ sources, particularly in urban areas [6]. For example, the CO_2_ emissions emitted from the urban area of Chennai, India were quantified by Kumar et al. [7], and the results showed that traffic contributed 29.7%. However, based on an overview of the experiences and results of transport CO_2_ reduction, Kelly and Zhu [8] suggested that reducing road transport emissions remained a major challenge in cities around the world. To develop and implement more practical traffic CO_2_ reduction measures in urban areas, investigating the effects of traffic CO_2_ emissions on CO_2_ fluxes and uncovering the characteristics of traffic CO_2_ emissions are in urgent need.

Investigating the influence of traffic on the CO_2_ flux is useful to uncover the role that traffic plays in urban carbon emissions. Coutts et al. [9] compared the CO_2_ flux between Preston and Surrey Hills and found that higher local traffic volumes contributed to higher CO_2_ fluxes even though the vegetation cover was higher in Surrey Hills. Gratani and Varone [10] compared five sites in the center of Rome. Their results indicated that traffic levels and tree coverage were both significantly correlated to CO_2_ concentrations. However, the comparability of CO_2_ fluxes and the influence of traffic on the CO_2_ fluxes among different cities or sites are still limited due to the distinct local climates and inconsistent observation periods. In addition, although traffic has a significant impact on CO_2_ fluxes and several mature micro-scale traffic CO_2_ emission estimation models, such as CMEM (Computer Program to Calculate Emissions from Road Transport, CMEM) [11], MOVES (Motor Vehicle Emission Simulator, MOVES) [12–14] and EMBEV (the Emission Factor Model for the Beijing Vehicle fleet) [15, 16], have been developed, most previous research have not applied these models to investigate the influence of traffic CO_2_ emissions on the CO_2_ flux [17].

Based on the national statistical data, many studies have revealed the annual variations of traffic CO_2_ emissions and proposed traffic CO_2_ reduction related policies at the national or regional level [18, 19]. China, with 10.29 billion tons CO_2_ emissions in 2014 [20], has become the largest CO_2_ emitter and has faced large pressure to reduce CO_2_ emissions. The CO_2_ emissions in the transport, storage and post sectors in China rapidly increased from 103.10 million tons in 1995 to 701.04 million tons in 2016 [21]. However, the energy consumption and CO_2_ emissions of the transport, storage and post sectors are not accounted separately, hardly at the city level or finer scale, and only contain the vehicles in operation, without the private transportation, in the statistical data in China. There are large amount of private vehicles in metropolitan areas. Taking Beijing as an example, the possession of private motor vehicles are 4.76 million, contributing 80.49% to the possession of motor vehicles [22].It’s necessary to conduct the traffic volume monitoring work at the city level and finer scale to estimate the urban traffic CO_2_ emissions and investigate the variation characteristics at various scales.

However, due to the mobility of the transportation, research on the traffic CO_2_ emissions on high spatial and temporal resolution are still a hot and difficult scientific topic. Ma et al. [23] estimated the passenger traffic CO_2_ emissions in urban Guangzhou in 2010 at the sub-district level by an improved bottom-up method and found an evident variation in the individual travel-related CO_2_ emissions between the workday (420 g per person) and the weekend (407 g per person). The higher daily traffic CO_2_ emissions on weekdays and the peak traffic CO_2_ hours were observed by Zhang et al. [24] in Nanjing in August 2013. Based on the taxi GPS data on the 1st November, 2012, Li et al. [25] found that the peak hour of taxi trips appeared at 10:00, 16:00 and 20:00, which showed different variations to the urban traffic demand. However, GPS data of private passenger cars, which contribute most to urban traffic CO_2_ emissions, are difficult to obtain because of privacy problems.

Traffic CO_2_ emissions are influenced by multi-factor interactions. Fontaras et al. [26] reviewed the influencing factors of traffic CO_2_ emissions and divided these factors into four categories, including vehicle characteristics, traffic and environmental conditions, driver related factors and vehicle certification tests. The results confirmed that traffic conditions and driving behaviors were highly influential. The road classes influence both the traffic conditions, including the average and maximum speeds, transient operation (accelerations-decelerations) and engine idling, and the behaviors of the drivers through the road capacity [27] and speed limit [28]. Nocera et al. [29] estimated the road transport CO_2_ emissions from different road classes, including motorways, primary roads and secondary roads, in Seville, Spain and compared the total CO_2_ emissions of the 32 road segments. The urban road classification system is somewhat different in different countries. In China, the urban road classification system includes four classes as follows: expressway, arterial road, sub-arterial road and collector road [30]. Since there are no statistical data of the energy consumption data of the urban traffic CO_2_ emissions at city level or finer scale and the traffic activity data at the road segment level can’t be obtained directly from the transportation management department in China, studies that investigate the traffic CO_2_ emissions of different classes of urban roads are insufficient.

To fulfill the shortcomings above, this study conducts a comparative analysis of the traffic CO_2_ emissions between two typical classes of urban roads on the high spatial and temporal resolution, the road link level and half-hour scale, and investigates their influence on the CO_2_ fluxes at two geographically proximate but in different types’ underlying urban areas in the Beijing Olympic Sports Centre Area, China. The Beijing Olympic Sports Centre Area is a pilot project for the construction of a low-carbon world city. It is bound to have a profound influence on the urban traffic CO_2_ reduction in Beijing and other cities in China and other developing countries. The two study areas include a mixed residential and commercial area with an intersection of urban arterial roads (DB) and an urban park area with an urban expressway segment (5R). The traffic are busy at urban expressways and arterial roads, and the mixed residential and commercial area is a kind of typical underlying urban areas in Beijing. In addition, the DB study area locates in the traffic restrict area and the 5R study area locates out of the traffic restrict area. The two study areas are geographically proximate and have consistent observation period of CO_2_ fluxes and meteorological factors. The traffic CO_2_ emissions are estimated by the localized MOVES model on the basis of traffic volume field monitoring data. These make the two sites more comparable. Comparing traffic CO_2_ emissions on various urban road classes and exploring their influence on CO_2_ fluxes at various underlying urban areas with similar geographies and climates but different anthropogenic activities, biogenic activities and different policy conditions are meaningful to help transportation department propose urban traffic CO_2_ reduction measures from the perspective of planning and designing. This paper will enhance the local understanding of the traffic CO_2_ emissions in metropolitan areas and provide scientific support to the accurate management of the CO_2_ reduction.

## Materials and methods

A comprehensive approach is established to analyze the role and characteristics of traffic CO_2_ emissions in various underlying urban areas. First, the boundary of the study area is defined by the Kormann and Meixner model (KM). Second, the CO_2_ flux data are obtained and processed through the ChinaFLUX data processing and quality control technology system. Then, the localized MOVES model is applied to estimate traffic CO_2_ emissions on the basis of the traffic volume field monitoring data. Finally, the multiple regression method is used to investigate the influence of traffic CO_2_ emissions on the CO_2_ flux.

### Definition of study area boundary

The spatial boundary of the study area is defined every 30 minutes by using the KM flux footprint model [31]. The KM function is as follows:

$$f\left(x,y,z_{m}\right)=f^{y}\left(x,z_{m}\right)D_{y}\left(x,y\right)$$ (1)

$$f^{y}\left(x,z_{m}\right)=\frac{1}{\Gamma \left(\mu \right)}\frac{\zeta^{\mu }}{x^{1+\mu }}e^{-\frac{\zeta }{x}}$$ (2)

where *f*(*x*, *y*, *z*_*m*_) (unit: m^-2^) denotes the total flux footprint, *f*^*y*^(*x*, *z*_*m*_) (unit: m^-1^) denotes the product of the crosswind-integrated footprint function, *D*_*y*_*(x*, *y)* (unit: m^-1^) denotes the crosswind dispersion function, $\mu =\frac{1+m}{r}$ is a constant, $\zeta =\frac{wz^{r}}{r^{2}q}$(unit: m) is the flux length scale, ω (unit: m^1-m^ s^-1^) denotes a constant of the wind speed power law profile, q (unit: m^1-n^ s^-1^) denotes a constant of the turbulent diffusion power law profile, *z* denotes the height from zero, and *r* denotes a shape factor (where *r* = *m* + 2−*n*, and *m* and *n* are indices of the wind speed and turbulent diffusion power law profiles, respectively).

To better understand the role of traffic CO_2_ emissions play in urban areas, we select two different types of urban areas where the urban roads of two classes are located. The 5th Ring Road study area (5R), an underlying urban park area, is located at 40° 01’ 35" N, 116° 23’ 29" E and adjacent to the 5th Ring Road, an urban expressway. The 5th Ring Road is not within the study area on a 30-minute scale through the entire year of 2014. To ensure the integrity of the road, this study defines the 5th Ring Road as the part that lies west to Lincui Road and east to Anli Road as the case road section in this study. More than 90% of the 5R study area is covered by plants. The Datun-Beichen West Road Intersection study area (DB), a mixed residential and commercial underlying area which is one of the most typical urban areas in Beijing, is located at 40° 00’ 06" N, 116° 22’ 45" E. The maximum footprint of the DB study area in 2014 covers 72.24 ha. There are ten buildings, and two urban arterial roads (Datun Road and Beichen West Road) with busy traffic flows and nearly 30% vegetation cover. The location proximity and climate similarity make the two underlying urban areas more comparable.

### Acquisition and processing of CO_2_ flux

The turbulent CO_2_ flux is measured by an open-air infrared CO_2_/H_2_O gas analyzer (Model Li-7500, LI-COR Inc., Lincoln, Nebraska, USA) and a three-dimensional ultrasonic anemometer (Model CSAT3A, Campbell Scientific Inc., Logan, Utah, USA). The gas analyzer is inclined to minimize solar radiation interference and facilitate the shedding of water droplets from the sensor lenses. All raw data (10 Hz) are recorded with a CR3000 (Model CR3000, Campbell Scientific Inc., Logan, Utah, USA).

Regarding the data post-processing and flux measurement quality control [32], 30-minute fluxes are derived and examined with the ChinaFLUX data processing and quality control technology system [33] as follows: outlier removal, time delay correction, planar fitting, high-frequency and low-frequency flux loss correction, ultrasonic virtual temperature correction and air density fluctuation effects on CO_2_ flux. We also examine the characteristics of the instruments and the reliability and quality of the CO_2_ flux measurements by calculating the power spectra and co-spectra. To reduce the interference of anomalous data, same-period observation data for precipitation, observation data beyond the threshold [–3, 3], the friction wind velocity beyond the threshold (determined by the method of Reichstein et al. [34]) and anomalous data are removed following the method of Papale et al. [35].

The non-linear regression method is used to interpolate missing data. A linear internal interpolation is used for short-term (<2h) missing data, and the Michaelis-Menten equation is used to interpolate long-term (≥2h) missing daytime data during the plant growing season. The longest stretches of missing data during the plant growing season are from 5:00 on the 20th May to 22:00 on the 22nd May at DB and from 13:30 on 27th June to 15:30 on 5th July at 5R. The time window is ten days. Night-time missing data are interpolated using the Lloyd and Taylor equation for CO_2_ flux [36, 37]. Because of the presence of non-biogenic CO_2_ fluxes in urban areas, the mean diurnal variation (MDV) method is also applied to fill data gaps, and a similar result is generated.

### Estimation of traffic CO_2_ emissions–localized MOVES model

Traffic flows at both the 5R and DB study areas were monitored from January to December in 2014. The main monitoring equipment include cameras (Sony HDR-CX510E, Sony Corporation, Tokyo, Japan) and a 7-meter-high tower. Three days, including two weekdays and one weekend day, are sampled every month. The traffic flow is monitored from 6:00 to 1:00 the next day. A man-machine interaction visual interpretation method is applied to classify the traffic flow into the following categories: large passenger vehicles, medium passenger vehicles, small passenger vehicles, heavy trucks, medium trucks, light trucks, ordinary buses, articulated buses, double-decker buses and other vehicles. The small passenger vehicles, which contribute more than 70% of the vehicles in the two study areas on the basis of the preliminary statistic, are classified into taxis and other small passenger cars. We also establish a group to validate the counting and classification results from the interpretation group. The random sampling validation, covering general and peak hours, weekdays and weekends for every month, is conducted. The classification results from the interpretation group and the validation group are compared. The data accuracy is greater than 95%.

Based the traffic flow monitoring and man-machine interaction visual interpretation data, the localized MOVES model is used to estimate traffic CO_2_ emissions every 30 minutes. The localization process of the MOVES model includes five steps: (1) Define the time span of the model. (2) Define the spatial boundary. Input the spatial boundary definition, which is defined by the KM model every 30 minutes of the study area. (3) Input the classified vehicle flow data. (4) Define the characteristics of the roads, including the road capacity, the limit speed of the road, the road grade and the signal light information. (5) Create the localization data management template. Create the temporary worksheet by the Project Data Manage of the MOVES model and localize the base emission factors, traffic condition factors and micrometeorological factors. We refer to Li et al. [17] for the base CO_2_ emission factors of a passenger car (with 7 seats or less, gasoline) in Beijing.

### Multiple regression

The multiple linear regression analysis method from SPSS 19.0 is used to quantitatively analyze the relationship between traffic CO_2_ emissions and the CO_2_ flux. The dependent variable is the CO_2_ flux, and the independent variables include radiation, temperature and traffic CO_2_ emissions. To enhance the comparability of the analysis, all variables are assessed every 30 minutes. According to the Administrative Measures on Heating in Beijing, the heating period starts on the 15th of November and ends on the 15th of March the next year. To better reveal the influence of traffic on the urban carbon cycle, the regression analysis is divided into two periods. One covers the entire year of 2014, and the other only contains the non-heating period.

## Results

### Annual CO_2_ emissions of the expressway and arterial road

The annual traffic CO_2_ emissions at 5R are more than ten times as much as the traffic CO_2_ emissions at DB. In 2014, the annual traffic CO_2_ emissions at 5R are 47,271.15 t and the annual traffic CO_2_ emissions at DB are 4139.19 t. The daily average traffic CO_2_ emissions at 5R are 129.51 t/d and the daily average traffic CO_2_ emissions at DB are 11.34 t/d. The traffic CO_2_ emissions of the urban expressway are higher than those of the arterial road in this study. Based on the intelligent transportation system data, Zhang et al. found that the daily average traffic CO_2_ emissions at urban arterials (7.44 t/d) were higher than urban highways (4.39 t/d) in Nanjing, China. Beijing, as the capital and the political, cultural, international communication and technology innovation center of China, has more traffic demand. Therefore, the daily average traffic CO_2_ emissions at the two sites in Beijing were higher than the daily average traffic CO_2_ emissions at urban arterials and urban highways in Nanjing. Large traffic volume at the ring expressway in Beijing led to more serious traffic congestion. The combination of the two factors caused more traffic CO_2_ emissions at the expressway than the arterial road in this study in Beijing. In Nanjing, the traffic activities mainly concentrate in the urban center areas where the traffic congestion is more serious. The urban arterial roads also concentrate in the urban center areas in Nanjing. Therefore, the adverse relationships of the traffic CO_2_ emissions between the expressway and arterial road are observed in Beijing and Nanjing. This phenomenon indicates that different cities have different demand for the design and construction of different classes of urban roads.

### Temporal variation characteristics

**Fig 1:**
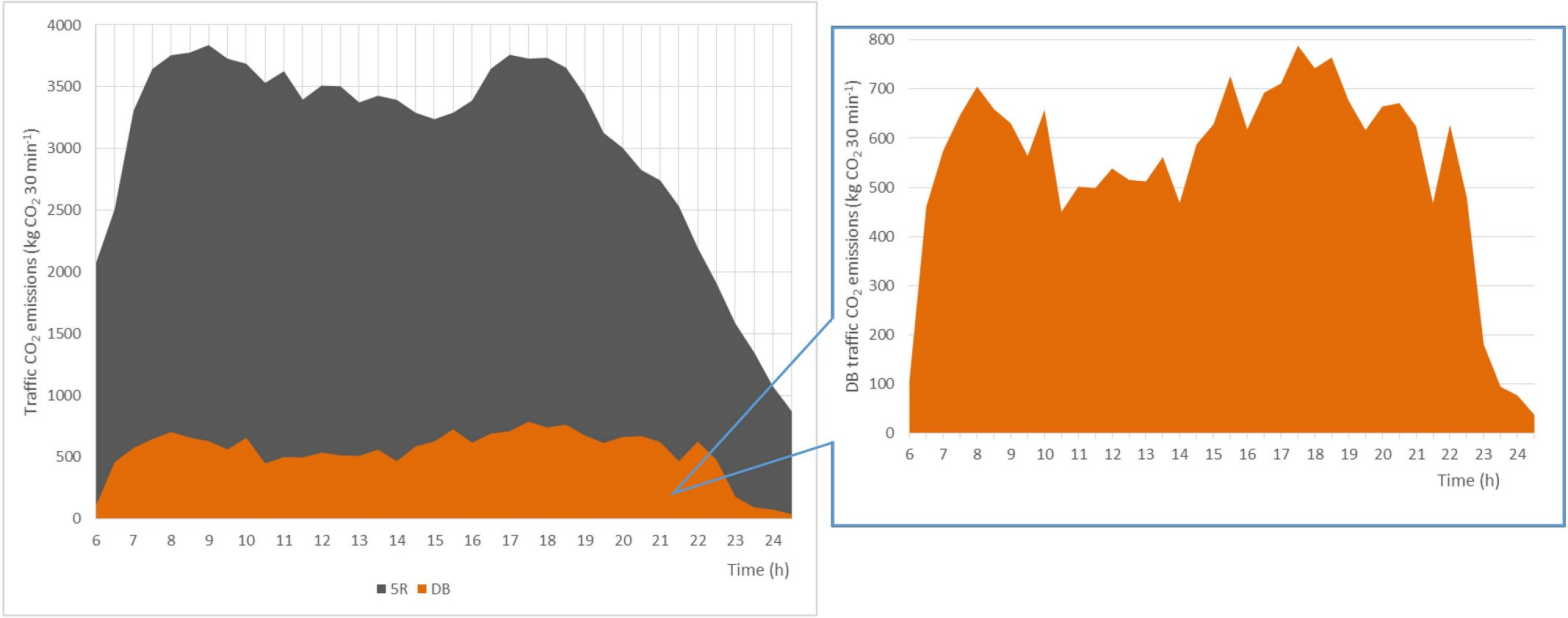
Comparison of diurnal variations of traffic CO_2_ emissions at 5R and DB.

Fig 1 shows the daily variation of the traffic CO_2_ emissions. The traffic CO_2_ emissions at 5R and DB both show a “double-peak” pattern, but the time and duration of the peaks of the traffic CO_2_ emissions are distinct. The morning peak of 3838.48 kg 30 min^-1^ appears at 9:00–9:30 at 5R, and the evening peak of 3759.63 kg 30 min^-1^ appears at 17:00–17:30. At DB, the morning peak appears at 8:00–8:30 with 705.14 kg 30 min^-1^, and the evening peak appears at 17:30–18:00 with 788.40 kg 30 min^-1^. The morning peak time of the traffic CO_2_ emissions at 5R appears 30 minutes later than at DB. The city center area of Beijing is north to the North 5th Ring Road and south to the South 3rd Ring Road. Most of the government agencies and departments, state-owned enterprises and government-affiliated institutions are located in the city center area of Beijing. These occupations need the employees to have a fixed and earlier office time. This can partly explain the earlier time of the morning peak of the traffic CO_2_ emissions at DB, located in the city center area of Beijing, than at 5R. In addition, the residents living in the city center area often have the habitats to take children to school by vehicles. The school hours in the morning starts earlier than commuting. It is also an important reason for the earlier morning peak time at DB.

The durations of the morning peak hours at 5R and DB are similar, but the duration of the evening peak hours at DB is two hours longer than at 5R. The morning peak of 5R lasts 2.5 hours from 7:30 to 10:00, and the morning peak of DB lasts three hours from 7:00 to 10:00. The evening peak of 5R lasts 2.5 hours from 16:00 to 18:30, and the evening peak of DB lasts 4.5 hours from 15:00 to 19:30. At DB, a small peak appears at 15:30–16:00, consistent with the end time of the school hours of the primary schools in Beijing. Picking up children from school to home by vehicles is an important reason for the small peak at 15:30–16:00. Later the commuting evening peak appears at 16:00–19:30. Therefore, the evening peak hours at DB appears earlier and lasts longer than at 5R. In addition, a sub evening peak appearing at 22:00–22:30 is also observed at DB, where located in the truck limit policy implemented area. The truck limit policy in Beijing regulates that no trucks can go into the area within the 5th Ring Road during 6:00–22:00. After 22:00, the traffic CO_2_ emissions at DB increase rapidly. It suggests that the truck limit policy has an important influence on the mitigation of the traffic congestion and the reduction of traffic CO_2_ emissions.

The average value and variation coefficient of traffic CO_2_ emissions by weekday and weekend at 5R and DB are shown in Table 1. The average daily traffic CO_2_ emissions at 5R are 131.74 t/d on weekdays and 126.33 t/d on weekends. The average daily traffic emissions at DB are 12.13 t/t on weekdays and 10.61 t/d on weekends. At 5R and DB, the average daily traffic CO_2_ emissions are both higher on weekdays than on weekends. Although the tail number limited measure on motor vehicles is implemented in Beijing on weekdays, the average daily traffic CO_2_ emissions on weekdays are still higher than on weekends. It indicates that the inelastic demands for traveling by vehicles, such as commuting and going to school, still dominate traffic CO_2_ emissions. Both on weekdays and weekends, the average daily traffic CO_2_ emissions at 5R are more than ten times higher than at DB. To overcome the influence from the mean values with large differences at DB and 5R, this study selects variation coefficients to make comparative analysis between the two case sites. The variation coefficients of traffic CO_2_ emissions are discrepant by weekday and weekend at 5R and DB. Both 5R and DB have higher variation coefficients of traffic CO_2_ emissions on weekends than on weekdays. At 5R, the variation coefficients are 17.80% on weekdays and 23.22% on weekends, respectively. At DB, the coefficients of variation are 3.05% on weekdays and 7.73% on weekends. The “rest effect”, less commuting traffic demand and more freedom on weekends are observed at 5R and DB. However, the variation coefficients of traffic CO_2_ emissions at 5R are much higher than those at DB on both weekdays and weekends. The traffic limited measures in Beijing have been carried out since 2008, and this policy is implemented on urban roads within the 5th Ring road (except the 5th Ring Road). Although the traffic CO_2_ emissions at 5R have also been influenced by the traffic limited measures, the influence is smaller than in the policy implementation area. Thus, more elastic and random travel demand leads to larger variation coefficients at 5R.

**Table 1:** Comparative analysis of traffic CO_2_ emissions of weekdays and weekends between 5R and DB.

Sites	Index	Weekday	Weekend	Weekday-Weekend
**5R**	Average daily traffic CO_2_ emissions (t/d)	131.74	126.33	5.41
	Coefficient of variation (%)	17.80	23.22	-5.42
**DB**	Average daily traffic CO_2_ emissions (t/d)	12.13	10.61	1.52
	Coefficient of variation (%)	3.05	7.73	-4.68

### Structural characteristics

**Fig 2:**
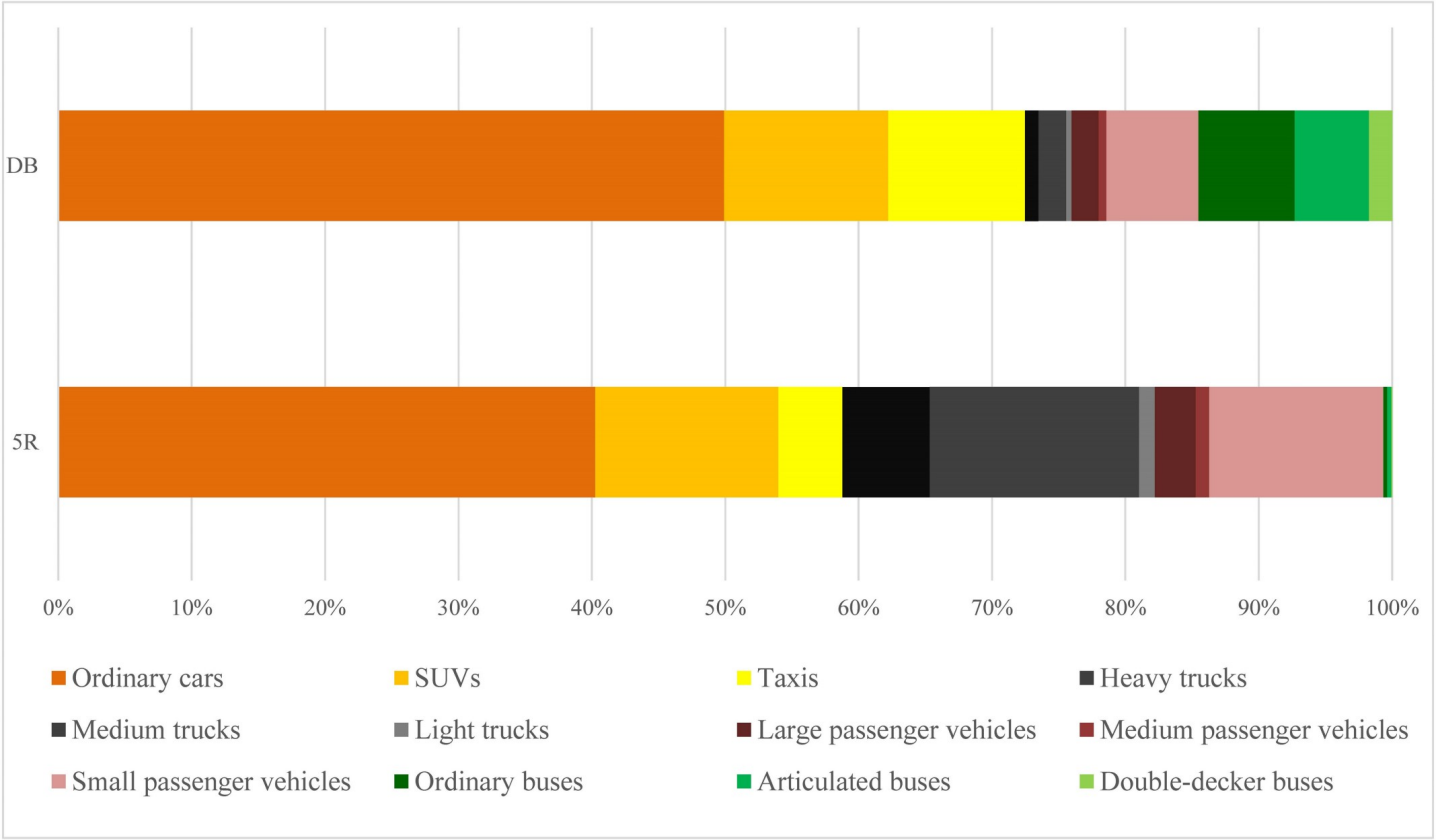
Structural characteristics of the traffic CO_2_ emissions at 5R and DB.

The structure of the traffic CO_2_ emissions at 5R and DB is shown in Fig 2. Most of the traffic CO_2_ emissions are derived from passenger vehicles at both 5R and DB. The contribution of passenger vehicles on the total amount of traffic CO_2_ emissions at 5R is 75.92%, and the contribution at DB is 81.99%. Among the passenger vehicles, private transportation dominates the traffic CO_2_ emissions in the metropolitan area. We subtract the traffic CO_2_ emissions derived from taxis, medium passenger vehicles and large passenger vehicles from the passenger vehicles, and assumes the results as the traffic CO_2_ emissions derived from private transportation. To some extent, it will underestimate the contribution from the private transportation since the traffic CO_2_ emissions derived from private trucks are not included. The private transportation contributed 67.02% and 69.14% to the total amount of the traffic CO_2_ emissions at 5R and DB, respectively. However, in China, the statistical data of the energy consumption of the transportation sector only contains the commercial vehicles at regional and city level. It will seriously underestimate the traffic energy consumption and CO_2_ emissions in the metropolitan areas where the private transportation have important influences on the traffic CO_2_ emissions.

Obvious differences in the structure of traffic CO_2_ emissions exist between the urban roads of the two classes. The contributions of taxis and buses, two typical types of public transportation, at DB are higher than that at 5R. The traffic CO_2_ emissions derived by taxis contributed 10.24% at DB and 4.80% at 5R. The traffic CO_2_ emissions derived by buses14.52% at DB and 0.67% at 5R. Most public transportation services are centrally provided in the city central areas, and people usually choose to take a bus or taxi when they travel relatively short distances. Therefore, the CO_2_ contributions of buses and taxis are both higher at the arterial road than the urban expressway in this study. Due to the truck limited measure, the contribution of trucks on traffic CO_2_ emissions at DB is lower than that at 5R. The CO_2_ emissions of trucks accounted for 23.41% at 5R and 3.49% at DB.

### Comparison of the effects of traffic CO_2_ emissions on the CO_2_ flux at different underlying urban areas with the urban roads of two classes

#### Temporal variation characteristics of the CO_2_ flux at different underlying urban areas

**Fig 3:**
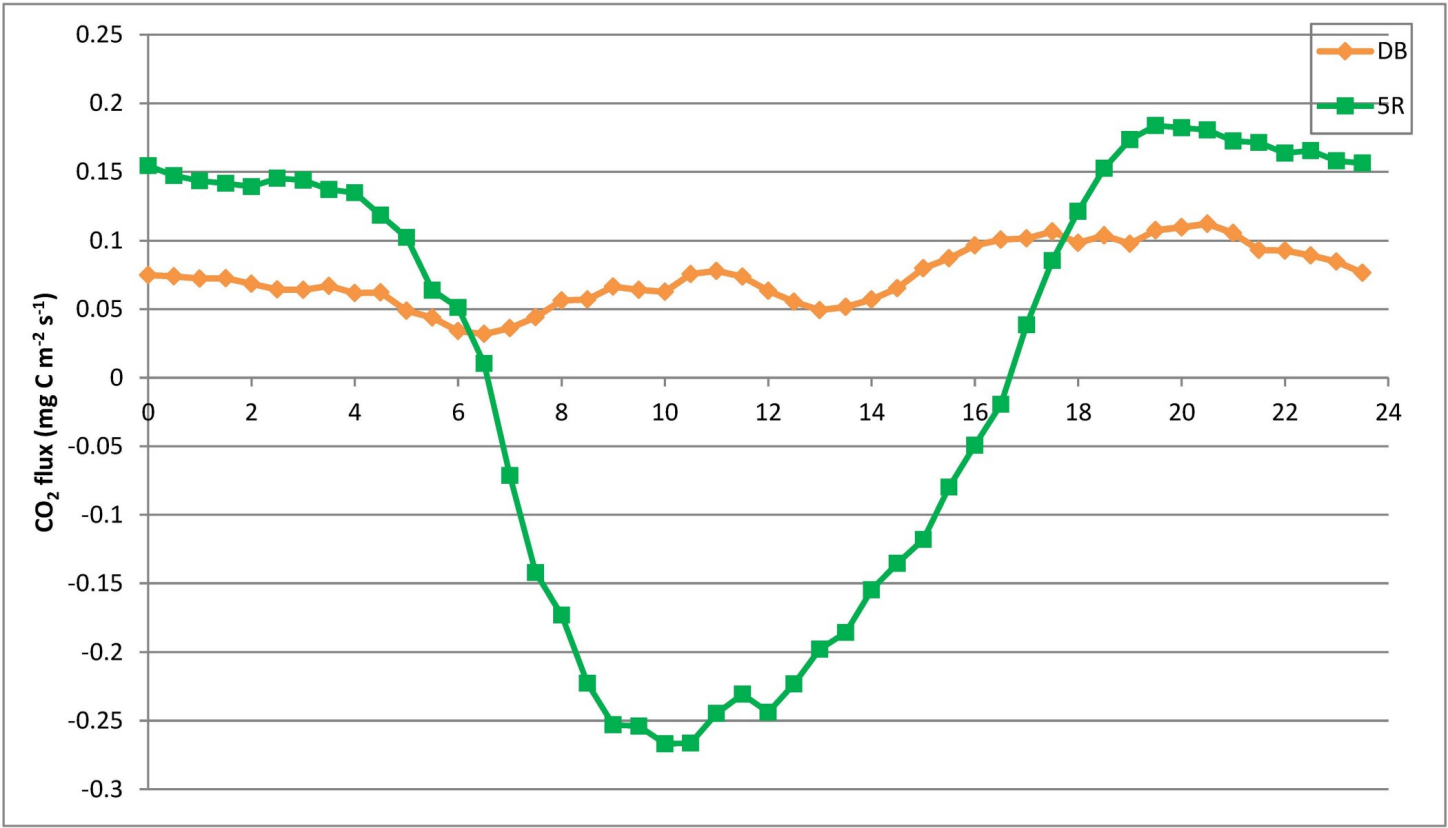
Diurnal patterns of the CO_2_ flux at 5R and DB.

The diurnal variation of the CO_2_ flux at 5R and DB is shown in Fig 3. The DB study area is always a CO_2_ source, whereas the 5R study area is a CO_2_ sink during the daytime but a source during the nighttime. The diurnal patterns of the CO_2_ flux at 5R and DB are distinct. At DB, the CO_2_ fluxes are all above zero and show a “double-peak” pattern. A small peak is observed during the daytime and an absolute peak appears at night. The small daytime peak (0.0779 mg C m^-2^ s^-1^) occurs during 11:00–11:30. The nighttime peak (0.1122 mg C m^-2^ s^-1^) occurs during 20:30–21:00 and is 0.03427 mg C m^-2^ s^-1^ (44%) higher than the daytime peak value. The diurnal pattern of the CO_2_ flux at 5R shows a “U-shape”. The valley value of -0.2668 mg C m^-2^ s^-1^ occurs during 10:00–10:30, and the CO_2_ flux values are below zero during 7:30–17:30. The CO_2_ flux at DB, the mixed commercial and residential urban area, varies less than that at 5R, the urban park area with a greater proportion of vegetation. During the daytime, vegetation plays a sink role by photosynthesis and leads to a decrement of the CO_2_ flux. During the nighttime, vegetation and soil release CO_2_ by respiration and lead to more CO_2_ emissions than the impervious ground. The proportion of vegetation cover at 5R is larger than that at DB, so the CO_2_ flux fluctuates more at 5R.

**Fig 4:**
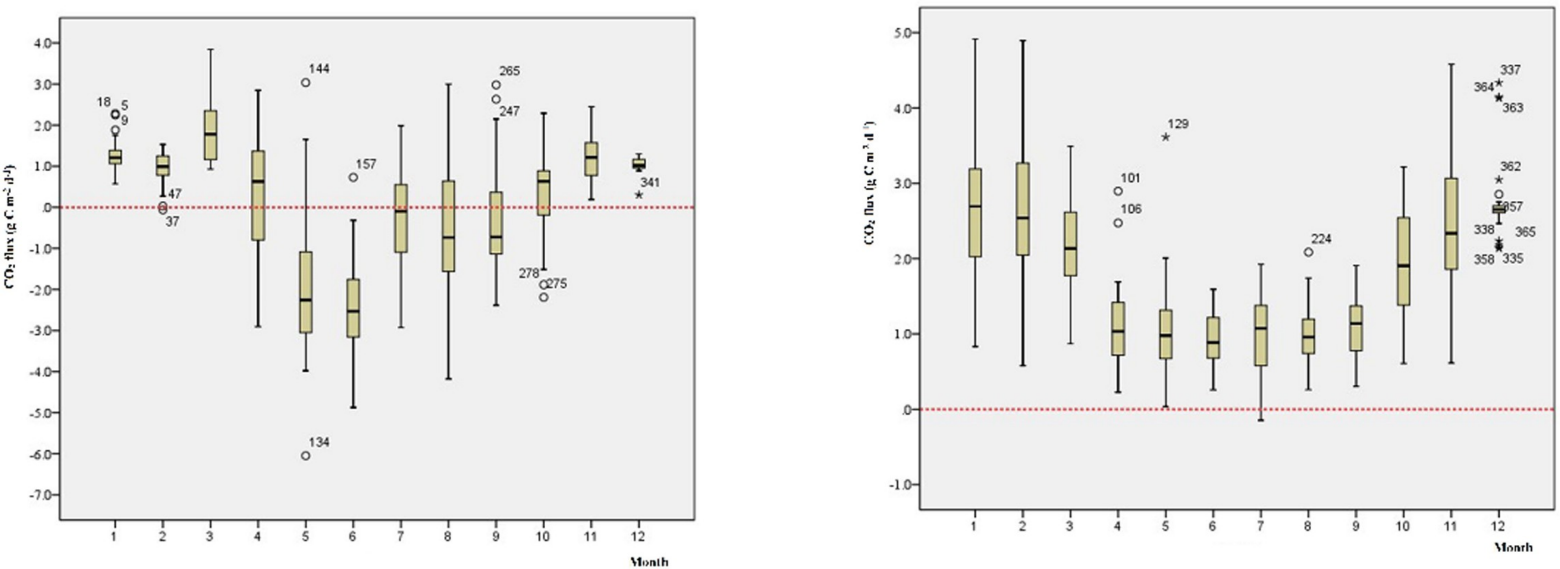
Monthly variations of the CO_2_ flux at 5R (a) and DB (b).

The monthly variation pattern of the CO_2_ flux at 5R and DB both show a “U-shape” (shown in Fig 4). The CO_2_ flux from January to March and from October to December in 2014 is higher, and from April to September, it is lower. However, the differences of the monthly variation between 5R and DB are reflected in three aspects as follows. (1) The CO_2_ flux at 5R is relatively lower than that at DB. In 2014, the annual average CO_2_ fluxes were 2.9747 g C m^-2^ d^-1^ and 0.3712 g C m^-2^ d^-1^ at DB and 5R, respectively. (2) The monthly average CO_2_ fluxes at DB are always above zero, whereas at 5R, the monthly average CO_2_ fluxes are below zero from May to September. (3) The CO_2_ flux changes more dramatically at DB from January to March and from November to December, covering the heating period in Beijing, generally consistent with the non-plant growing season. However, at 5R, the CO_2_ flux changes more dramatically from April to October, covering the plant growing season.

#### Influences of traffic CO_2_ emissions on the CO_2_ flux

Based on the distinctive characteristic of the CO_2_ flux between the plant growing season and non-plant growing season as mentioned above, this study not only conducts a multiple regression analysis for the entire year, but also extracts the data of the plant growing season to further investigate the effects of traffic CO_2_ emissions on the CO_2_ flux during the plant growing season. The stepwise regression results are shown in Table 2. At a significance threshold of sig. < 0.001, the models of both 5R and DB for the entire year of 2014 pass the significance test, and the R values are 0.38 and 0.66 at DB and 5R, respectively. Both models of 5R and DB during the plant growing season pass the significance test, and the R values of 0.78 and 0.31 at 5R and DB, respectively. Although the R values are relatively low, related research results, such as the model R value of 0.3 in Hiller et al. [38] and of 0.5 in Contini et al. [39], show that the fitting of the models in this study is acceptable.

**Table 2:** Comparison of regression results of different urban underlying areas by the entire year and plant growing season in 2014.

Site	Variance	Entire year		Plant growing season	
		Non-standard coefficient	Sig	Non-standard coefficient	Sig
DB	(Constant)	0.123	0.000	0.095	0.000
	Temperature	-0.003	0.000	-0.002	0.000
	Radiation	-6.845×10^−5^	0.000	-6.213*10^−5^	0.000
	Traffic CO_2_ emissions	7.931×10^−8^	0.001	5.582*10^−8^	0.001
5R	(Constant)	-0.078	0.000	0.029	0.395
	Radiation	-0.001	0.000	-0.001	0.000
	Temperature	0.004	0.000	0.005	0.011

For the entire year, the temperature (sig. < 0.001), radiation (sig. < 0.001) and traffic CO_2_ emissions (sig. = 0.001) all have significant effects on the CO_2_ flux at DB; the radiation (sig. <0.001) and temperature (sig. < 0.001) have significant effects on the CO_2_ flux at 5R. The regression results show that the influence of traffic CO_2_ emissions is not significant at 5R, but that the influence of traffic CO_2_ emissions is significant at DB. It suggests that traffic CO_2_ emissions play an important role at the mixed commercial and residential urban underlying area with urban arterial roads, though the traffic CO_2_ emissions at 5R are more than ten times than those of DB. This phenomenon is related to the higher proportion of vegetation cover at 5R (more than 90%) than at DB (27.53%). With a larger proportion of vegetation cover, the 5R study area has more CO_2_ sequestration. The coefficients of radiation are negative at both 5R and DB. When the radiation increases, the CO_2_ flux decreases. In addition, the variation of the CO_2_ flux caused by one unit change of the radiation is larger at the 5R (-0.001) than at DB (-6.845×10^5^). It indicates that vegetation is important for the CO_2_ sink by the photosynthesis, and that vegetation at the urban park area can compensate for the large amount of CO_2_ emissions derived from traffic.

## Conclusions

Based on the 36-day classified traffic flow monitoring and interpretation work on the urban roads of two classes and one-year CO_2_ fluxes and micro-meteorological factors at two different types of underlying urban areas in Beijing, this study uncovers the differences of the traffic CO_2_ emissions between the urban roads of two classes from the perspective of temporal variation, structural characteristics and their influences on the CO_2_ flux. The following conclusions can be drawn:

Obvious differences exists in the temporal variation characteristics of the traffic CO_2_ emissions between the expressway and arterial road at various temporal scales. At the annual scale, the traffic CO_2_ emissions at the expressway (5R, with 47271.15 t) are more than ten times than those at the arterial road (DB, with 4139.19 t). At the week scale, “rest effect” is observed at the urban roads of two classes, and the elastic is stronger at the expressway. At the daily scale, the differences of the peak time and duration between the urban roads of two classes suggest that the traffic CO_2_ emissions of urban arterial roads are influenced by the travel demand derived of urban life and the existing traffic limit policies.

The traffic CO_2_ emissions derived from passenger vehicles play a predominant role in the CO_2_ emissions of urban on-road transportation, and more traffic CO_2_ emissions are derived from the private transportation than those of the public transportation at the urban roads of the two classes in this study. However, significant differences existed in the structural characteristics of traffic CO_2_ emissions between 5R and DB. The passenger transport contributed nearly all the traffic CO_2_ emissions at DB, but the traffic CO_2_ emissions derived from freight transport play an important role at the expressway 5R (23.41%). The buses contributed more traffic CO_2_ emissions at the arterial road (14.52%) than at the expressway (0.67%). The concept of shared mobility provides a path to improve the efficiency of the private passenger transport, which accounted for most of the traffic CO_2_ emissions at urban areas, and to reduce the traffic CO_2_ emissions. The promotion of the shared mobility demands the formulation and improvement of the related laws and regulations to ensure the safety of drivers and passengers and regulate the use and park of the shared cars.

Although the traffic CO_2_ emissions at the expressway are more than ten times than those of the arterial road, the influence of traffic CO_2_ emissions on the CO_2_ flux is significant at the commercial and residential mixed underlying urban areas with arterial roads but not significant at the urban park area with expressway in this study. The results indicated that the green space along the urban roads had important effects in the CO_2_ absorption. Increasing the design and construction of the green space along the urban roads with busy traffic flow can mitigate the direct CO_2_ emissions produced by the urban transportation.

## Discussions

To estimate the local traffic CO_2_ emissions more accurately, this paper localizes the MOVES model mainly from the aspects of the base emission factors, micro-meteorological factors and road segment characteristics. However, this paper only modified the base emission factors of the passenger cars with seven seats or less, which accounted for more than 80% of the total traffic volume of the study areas. Due to a lack of CO_2_ emission factors for other types of vehicles proposed by a national or regional authority in China, this paper directly uses the base emission factors, embedded in the MOVES model, of other vehicles. It will lead to the uncertainty of the estimation of the total amount of traffic CO_2_ emissions in this study.

Since local traffic detection data cannot be obtained directly from the transportation management department in China, we conduct traffic field monitoring work three days per month in 2014 at the two case sites in Beijing. However, due to the high economic and labor costs of the collecting and interpreting process, the traffic CO_2_ emissions on the days without traffic field monitoring data are substituted by the traffic CO_2_ estimation results of the adjacent workday or weekend. This substitution process will lead to uncertainty in the annual total traffic CO_2_ emissions values.

Due to the data limitations, this study only compare the traffic CO_2_ emissions between the urban roads of two classes. Given the various classes of urban roads and the various types of underlying urban areas, research from a study of one single fixed point site to a comparison study and a transect analysis are of great significance to further uncover the role of traffic in the urban CO_2_ emissions and to provide scientific references to reduce the traffic CO_2_ emissions at urban areas and to construct the low-carbon transportation system.

## Supporting information

S1 Data (RAR)

S1 File (DOCX)
