# Supplementary material for: Comparative analysis of the CO2 emissions of expressway and arterial road traffic: A case in Beijing
Source: PLoS One. 2020 Apr 14;15(4):e0231536. doi: 10.1371/journal.pone.0231536 (PMC7156062; doi:10.1371/journal.pone.0231536)
Supplement: S1 File — (DOCX) [file pone.0231536.s002.docx]

**Vehicle classification method applied in this study**

| Passenger vehicle | Large passenger vehicle | length≥6 m or number of passengers≥20 | |
| --- | --- | --- | --- |
|  | Medium passenger vehicle | length＜6 m, 9＜number of passengers＜20 | |
|  | Small Passenger vehicle | Length<6 m,  Number of passengers≤9 | Taxis  Others |
| Truck | Heavy-duty truck | Length≥6 m or mass≥12000 kg | |
|  | Medium-duty truck | Length≥6 m, 4500 kg≤mass＜12000 kg | |
|  | Light-duty truck | Length<6 m, mass<4500 kg | |
| Bu | Ordinary bus | Single bus with 2 doors | |
|  | Articulated bus | Articulated bus with 3 doors | |
|  | Double-decker bus | Double-decker bus | |
| Others | Other types that appeared less often in the study area | | |
